# Case Report: Safety and efficacy assessment of teclistamab in relapsed/refractory amyloidosis: a case study

**DOI:** 10.3389/fonc.2025.1664171

**Published:** 2025-09-18

**Authors:** Zhe Chen, Zhijian Zhang, Jieni Yu, Leihua Fu, Jiaping Fu, Weiying Feng

**Affiliations:** Department of Hematology, Shaoxing People’s Hospital, Shaoxing, China

**Keywords:** amyloidosis, teclistamab, relapsed/refractory, bispecific antibody, safety and efficacy

## Abstract

Systemic light chain amyloidosis (AL) is an uncommon disorder of clonal plasma cells, marked by the creation of amyloid-forming immunoglobulin light chains that result in amyloid fibril formation and deposition, causing dysfunction in multiple organs. There is no established treatment protocol for patients with relapsed or refractory AL. Teclistamab is a bispecific antibody targeting B-cell maturation antigen (BCMA) and is approved for treating relapsed or refractory multiple myeloma (MM). The objective of our study was to report real-world data on the safety and efficacy of teclistamab in patients with relapsed/refractory AL.

## Introduction

Systemic light chain amyloidosis (AL) is a condition involving plasma cells that leads to the misfolding and extracellular accumulation of immunoglobulin light chains, leading to harm in various organ systems such as the heart (60%), kidneys (50% - 80%), liver (25% - 60%), soft tissue (40%), peripheral/autonomic nervous system (15%), and gastrointestinal tract (5%) (1). AL can lead to serious dysfunction in multiple organ systems, and approximately 30% of patients may die within six months of being diagnosed (2).

Therapeutic strategies for AL aim at the clonal plasma cells and have historically used anti-plasma cell agents effective in multiple myeloma (MM) treatment. The sole Food and Drug Administration (FDA)-approved treatment plan for AL, which is also the standard initial therapy, consists of daratumumab, cyclophosphamide, bortezomib, and dexamethasone. Despite enhancements in therapies that include bortezomib, the rates of achieving a complete hematologic response are still below expectations. There is a high rate of early mortality, with outcomes differing according to the degree and seriousness of organ involvement, and treatment-related toxicity is common (2). And Patients with relapsed AL who are refractory to daratumumab lack a standard treatment protocol and usually receive combination therapies based on MM treatment models (3, 4).

As of now, the FDA has granted accelerated approval to B-cell maturation antigen (BCMA)-targeting bispecific antibodies (bsAbs), specifically teclistamab, for use in relapsed/refractory myeloma. As a bispecific antibody, Teclistamab redirects T-cells by targeting CD3 on T-cells and the BCMA on plasma cells. Teclistamab produced profound and sustained responses in individuals with relapsed or refractory (R/R) MM during the MajesTEC-1 trial (5). BCMA-targeting bsAbs are an enticing treatment option for AL due to several reasons: (a) rapid attainment of profound hematologic responses in myeloma is crucial for organ response in AL; (b) there is a reduced occurrence of severe cytokine release syndrome (CRS) compared to other T-cell redirecting treatments like chimeric antigen receptor T-cell therapy. No clinical trials have yet provided data on the safety and efficacy of teclistamab in AL. This report discusses a case involving a patient with R/R AL who received successful treatment using the bispecific antibody teclistamab.

## Case report

**Figure 1:**
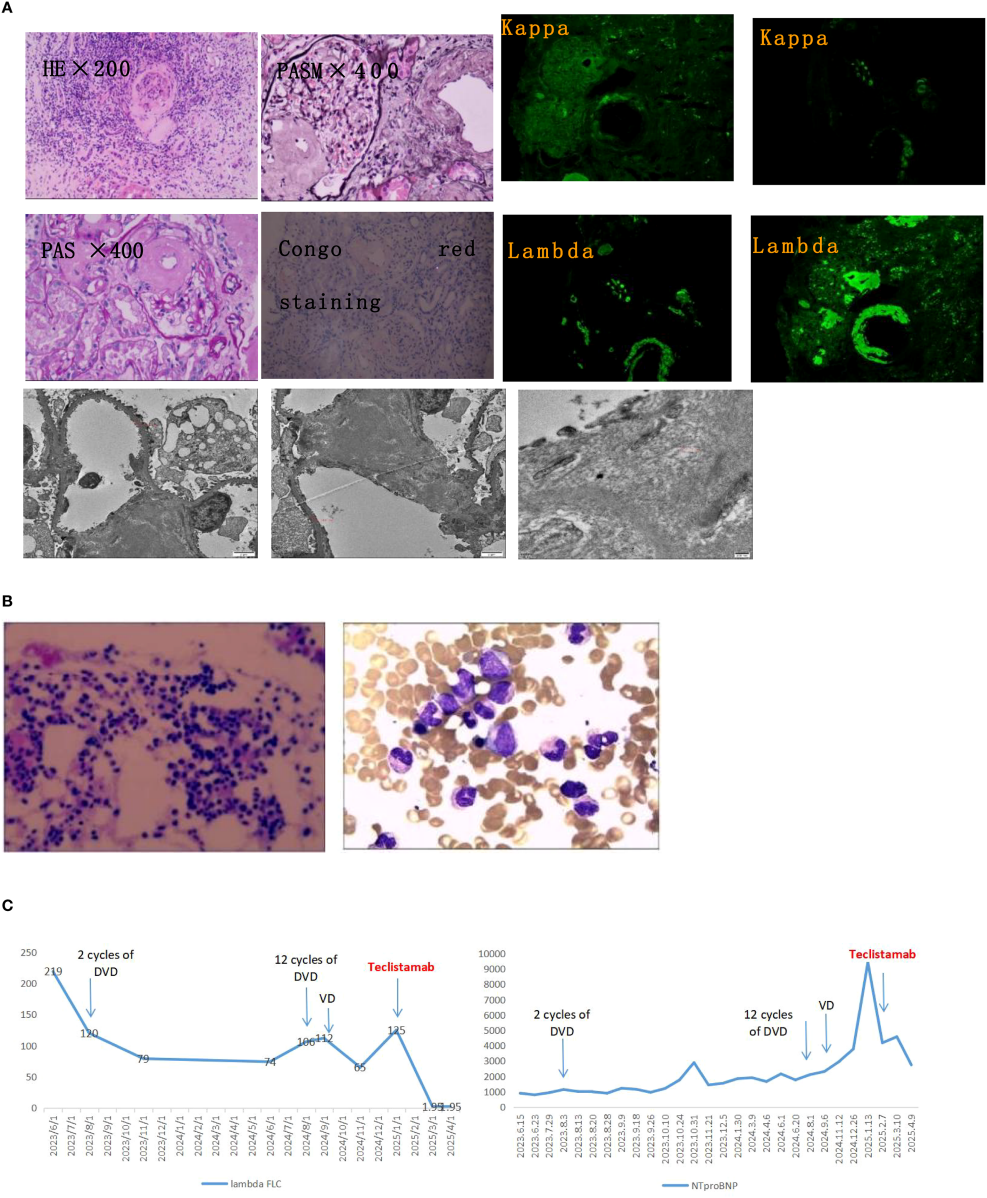
Diagnostic pathology and longitudinal treatment response. **(A)** Renal pathological assessment. Upper left: Congo red staining under polarized light shows characteristic apple-green birefringence, confirming amyloid deposits. Upper right: Immunofluorescence demonstrates strong lambda light chain restriction (+++) with kappa light chain negativity, diagnostic for AL-lambda amyloidosis. Bottom: Transmission electron microscopy reveals randomly oriented, non-branching amyloid fibrils measuring 8–10 nm in diameter. **(B)** Bone marrow evaluation. Wright-Giemsa staining shows no evidence of myeloma cell infiltration or clonal plasma cell expansion. **(C)** Serial biomarker trends. The vertical dashed line denotes initiation of teclistamab therapy. Lambda free light chain (λ FLC) levels (mg/L, left axis) rapidly declined to undetectable ranges post-treatment. Concurrently, NT-proBNP levels (pg/mL, right axis) showed substantial reduction, reflecting cardiac functional improvement. DVD, daratumumab-bortezomib-dexamethasone; VD, bortezomib-dexamethasone; NT-proBNP, N-terminal pro-brain natriuretic peptide.

A 64-year-old man with a history of hypertension was admitted to the hospital on June 23, 2023, due to persistent lower limb swelling that had lasted over six months. The examination conducted during hospitalization indicated Hemoglobin (119g/L, 130-175g/L) and Serum creatinine (115.2μmol/L, 59-104μmol/L), serum protein electrophoresis and immunofixation electrophoresis were notable for a monoclonal IgA λ immunoglobulin, serum kappa FLC was 31.2 mg/L, lambda FLC was 219 mg/L with a kappa to lambda ratio of 0.14. 24-hour proteinuria: 4.1g/d (0-0.15g/d). Tspot, thyroid function, glycosylated hemoglobin, ANA, ANCA, anti-glomerular basement membrane antibody, NTproBNP, troponin and CMV-DNA were normal. Pathological examination following a renal biopsy showed AL-type amyloidosis in the kidneys (**Figure 1**). The cardiac MRI revealed heart involvement and an enlarged left atrium, with diffuse thickening of the left ventricular septal wall. According to the modified European criteria, by the Mayo 2012 criteria, he was at stage II, with renal stage II AL.

He began treatment with 2 cycles of DVD (daratumumab, bortezomib, and dexamethasone), the lambda FLC decreased to 120 mg/L, yet there was no improvement in renal function (Serum creatinine rose to 162 μmol/L) and NTproBNP (1132 pg/ml, 0-125pg/ml). 9 cycles of treatment with DVD were completed, the lambda FLC decreased to 74 mg/L, yet there was no improvement in renal function (Serum creatinine rose to 272 μmol/L) and NTproBNP (2154 pg/ml). And the patient’s immunofixation electrophoresis remained positive. During the 12th cycle of the DVD regimen, he relapsed with worsening renal function (Serum creatinine rose to 333 μmol/L) and NTproBNP (2307 pg/ml), and rising lambda FLC (106 mg/L). The medication also caused the patient to develop peripheral neuropathy as an adverse reaction. The patient felt numbness and discomfort in their limbs, along with occasional nighttime pain. Following this, the treatment was altered to a VD regimen (bortezomib and dexamethasone) for 5 rounds. However, the treatment goal was not met. The patient’s immunofixation electrophoresis continued to be positive, and there was a decline in renal function (Serum creatinine rose to 365 μmol/L), an increase in NTproBNP (9421 pg/ml), and elevated lambda FLC (125 mg/L). We once more examined the bone marrow and did not find a restricted plasma cell clone (**Figure 1**).

Due to his resistant disease, the patient received teclistamab, which the FDA (5) has approved for use in MM after four lines of treatment. At the beginning, the serum creatinine level rose to 260μmol/L, NTproBNP was measured at 4177 pg/ml, and lambda FLC was 125 mg/L. He managed the gradual increase (teclistamab 0.06mg/kg d1, 0.3mg/kg d2, 60mg d8,15,22) in dosing without developing CRS or immune effector cell-associated neurotoxicity syndrome (ICANS). The encouraging news is that after completing one cycle, both serum immunofixation and lambda FLC were negative. The patient reached a state of complete remission. Subsequently, he switched to receiving subcutaneous injection every two weeks starting from cycle 3 (teclistamab 60mg q2w). Throughout the treatment, no further adverse reactions were observed, and the patient’s cardiac issues (NTproBNP was measured at 2738 pg/ml) improved considerably (**Figure 1**). Regrettably, the patient’s kidney function did not enhance as expected and continued to decline, the serum creatinine level rose to 362μmol/L. It might be that the kidneys’ structure is impaired, leading to difficulties in regaining their functions. The medical examination during the hospitalization on April 9th, 2025, indicated Hemoglobin (106g/L) and Serum creatinine (362μmol/L), serum immunofixation and lambda FLC were negative, 24-hour proteinuria: 3.2g/d, NTproBNP (2738 pg/ml).

This patient was generally hard to manage because of relapse, refractory, and declining cardiac and renal function. The use of teclistamab led to a swift hematologic and clinical response, with the patient maintaining a lasting response to the treatment. Trials generally exclude patients who have severe kidney failure along with MM or AL. Patients with severe kidney dysfunction frequently need hemodialysis or peritoneal dialysis, which may also impact the effectiveness of the medication. Perhaps it is also related to the fact that the drugs are difficult to improve the patient’s kidney function. This research is based on a single case report, which is limited by its small sample size, and its findings may not apply to all patients with R/R AL. There is still debate over the follow-up consolidation therapy for R/R AL patients who have achieved complete remission. Additional research involving more patients and extended follow-up periods is necessary to verify the effectiveness and safety of anti-BCMA bispecific antibodies in R/R AL.

## Data Availability

The raw data supporting the conclusions of this article will be made available by the authors, without undue reservation.
